# The new prognostic-therapeutic index – an easy method of establishing surgical indication in the pathology of the diabetic foot

**Published:** 2014

**Authors:** F Bobircă, E Catrina, O Mihalache, D Georgescu, T Pătrașcu

**Affiliations:** * “Dr. I. Cantacuzino” Clinical Hospital, “Carol-Davila” University of Medicine and Pharmacy, Bucharest

**Keywords:** diabetes mellitus, diabetic foot, prognostic-therapeutic index

## Abstract

**Hypothesis and aim.** The large number of invalidating surgical interventions in patients suffering from lesions of the diabetic foot, the late recognition of the lesions and sometimes the wrongful interpretation of their severity, have made necessary a multi-parameter study of these types of patients and the elaboration of a therapeutic-prognostic index to guide the physician in adopting the adequate method of treatment.

Starting with the therapeutic-prognostic index imagined by professor Traian Patrascu, we have elaborated a new therapeutic prognostic index, by adding new, statistically significant parameters, for the purpose of facilitating the surgical indication, depending on the lesion type.

**Methods.** A number of 929 patients who were admitted at the Surgery Clinic of the “Dr. I. Cantacuzino” Hospital, between January 2013 and June 2014, have been analyzed, of whom 450 were evaluated retrospectively and 479 prospectively.

**Results.** The new therapeutic prognostic index has been calculated for the retrospective lot, resulting into a concordance between the actual surgical intervention and the prognostic index of 79.4% and, for the patients evaluated prospectively, we have found a confirmation of the relation of 82.6% between the performed surgical intervention and the forecasted surgical intervention, by calculating the index.

**Discussion.** The new therapeutic-prognostic index represents an easy method of establishing the therapeutic conduct of the patient suffering from lesions of the diabetic foot.

It is of major use in preventing the execution of such surgical interventions that may be disproportionate compared to the severity of the lesions, especially in facilities where the pathology of the diabetic foot is less known.

## Introduction

The diabetic foot is a complex pathologic entity that includes all the modifications which occurred at the foot level during the course of evolution of the diabetic disease (A. J. M. Boulton).

As an impairment for which the diagnostic is usually performed late, many times the recognition of these types of lesions is done at a phase in which the only surgical solution is represented by a major amputation of the pelvic limb [**1**,**2**,**6**,**7**]; therefore, a clarification regarding the assessment of the nature of these types of lesions, as well as the surgical interventions is absolutely necessary, also because the majority of patients suffer from other diseases that influence the general condition of the patient as well, and to which an infection factor is added.

As a less known pathology, the diabetic foot requires a multidisciplinary approach [**1**,**3**,**4**] as a therapeutic attitude, therefore clinical and paraclinical parameters that quantify the condition of the vascular bed, the loss of sensitivity, the patients’ associated tares, the condition of the skeletal system, of teguments are very important and must be studied, and last but not least, the compensation of the diabetic disease, which is an essential criterion on the balance of which the healing of these types of lesions depends.

Based upon the therapeutic-prognostic index elaborated by professor Traian Patrascu [**1**], we have imagined a new therapeutic prognostic index, adapted to recent investigation methods.

The therapeutic-prognostic index includes a parameter scale [**5**] representing the main risk factors for the diabetic foot lesions to which given values are assigned, depending on the severity [**1**]. It has been elaborated by using a specific methodology of calculus, applied on a database, developed by means of selecting patients with diabetic foot lesions on both clinical and paraclinical criteria [**1**].

The results of applying the methodology, under the shape of the therapeutic-prognostic index are distributed on a scale of 1 to 10, the value thusly quantified being directly proportional to the severity of the lesions. Therefore, there are two categories of results: with a value of less than 5, the recommendation for such cases being a minor surgical intervention (simple drainage incisions, amputation of phalanges, toes amputations, foot cross-metatarsal amputations), and for values of over 5 of the index, the indication being of major amputation (calf or thigh).

Starting from both the retrospective and the prospective research of the patients lot, after analyzing the clinical as well as the paraclinical parameters, we have elaborated this new therapeutic prognostic index, for the purpose of improving the results of the diabetic foot surgery, thus avoiding repeated operations, disproportionate by comparison to the extent of the lesion, in an attempt not to transform this type of patient into a permanently socially dependent person. 

The large variety of diabetic gangrene lesions that require different therapeutic decisions represents the main reason for identifying an efficient method of evaluation and surgical treatment.

## Methods

A number of 929 patients suffering from the following affections were admitted at the Surgery Clinic of the “Dr. I. Cantacuzino” Hospital, between January 2013 and June 2014, of whom, 450 were evaluated retrospectively and 479 prospectively:

• Diabetes mellitus type I or type II;

• Diabetic foot lesions;

• On a predominantly arteriopathic or neuropathic field; 

• With or without associated impairments: cardio-vascular, renal, ocular;

• With or without septic status;

A monitoring scheme, including the following parameters for the evaluation of the diabetic foot, has been created for these patients: 

o Gender of the patient;

o Age; 

o Type of diabetes; 

o First appearance of diabetes;

o Way and degree of compensation of the diabetes;

o Degree of arteriopathic/ neuropathic impairment; 

o Sepsis association, anemia;

o Association with cardio-vascular, ocular, renal impairment;

o Type of surgical intervention;

o Post-surgery evolution/ re-interventions 

The parameters which were included in the evaluation table for the diabetic foot patient are the following:

**Table 1:** Diabetic foot evaluation chart

1. Identification Data	5. Complications of diabetes
- Age	- type (arteriopathy, neuropathy, nephropathy, etc.)
- Gender	- diagnostic (clinical, para-clinical)
- Background	- **Neuropathy:** sensitivity tests in tactile, thermal, vibration, pain; ROT, electro-diagnostic test, abnormal testing of vegetative functions (sinus arrhythmia, decrease of sweating, increase of pupil latency), simple radiography of the foot
- Social status	- **Arteriopathy:** - clinical tests; taking the pulse, modifications in the tegument coloring, presence of edema, temperature gradient, modifications of the tegument (atrophy and thinning of the tegument, absence of hairiness, onychodystrophy); venous filling time
2. Environmental and individual factors	- non-invasive testing: Doppler arterial, venous, ankle-brachial index
- Toxic emission – smoking	- invasive testing: Arteriography, MR Angiography, CT Angiography
- Consumption of alcohol	6. Type of Surgical Intervention
- Consumption of drugs	- incisions, debridement
- Body mass index	- minor amputations
3. Comorbidities	- major amputations
- Type	7. Associated Complementary Methods
- Phase	8. Post-Surgery Evolution
- Treatment	9. Re-interventions
4. Sugar Diabetes	10. Re-admissions
- Type	
- Seniority	
- Treatment	
- Diet	

We have extended the therapeutic-prognostic index by adding new parameters, after making a preliminary test of their statistical significance, one of them being correlative and by analyzing the extended therapeutic-prognostic index on a wider range of patients, to ensure an increase in the statistical significance of the study. 

The two parameters that were added to the therapeutic-prognostic index are the following:

• **Surgical interventions for the diabetic foot in antecedents**, occupying position IX in the new index.

• **Glycaemia + leukocytosis**, being assigned the Position X.

The parameter: **surgical interventions for the diabetic foot in antecedents**

The analysis is done according to the following:

- No intervention; 

- One intervention

 - Two interventions

- More than two interventions

• Surgical interventions in antecedents signify an inadequate compensation of the sugar diabetes, lack of compliance, precarious biologic terrain and **it is statistically associated with an evolution towards major amputation.**

• Assigned values:

 - No intervention = 0 p

 - One intervention = 0.2 p

- Two interventions = 0.4 p

- More than two interventions = 0.6 p

The parameter: **glycaemia + leukocytosis**, presents a statistical significance and represents an expression of the inter-relation between the two composing parameters.

**Table 2:** Parameter: glycaemia + leukocytosis

Glycaemia (mg/dl)	Leukocytes(/mm3)	Assigned Value (points)*
115-150	11,000-15,000	0.2
150-200	15,000-20,000	0.4
200-300	15,000-20,000	0.6
300-400	15,000-20,000	0.8
>400	>20,000	1
*All the values falling outside these intervals are evaluated at 0 p

The devices necessary for calculating the new therapeutic-prognostic index are the following: the ankle-brachial index measurement device and the neuropathy evaluation kit. 

The new therapeutic-prognostic index consists of the following parameters:

**I. Gender ** Male = 0.4 points

Female = 0.2 p

**II. Age of the patient ** 20-29 years old = 0.1 p

30-39 years old = 0.2 p

40-49 years old = 0.4 p

50-59 years old = 0.6 p

60-69 years old = 0.8 p

70-.... years old = 1 p

**III. Duration of Diabetes ** 1 -5 years old = 0.2 p

6-10 years old = 0.4 p

11-15 years old = 0.6 p

16-20 years old = 0.8 p

20-.... years old = 1 p

**IV. Type of lesion **

-simple neuropathic ulceration = 0.2 p

-deep neuropathic ulceration = 1 p

-ulceration with extensive humid gangrene = 2.4 p

-dry gangrene of the toes = 0.2 p

-humid gangrene of the toes = 1.1 p

-extensive humid gangrene = 2.4 p

-deep abscess of the foot = 0.4 p

-necrotizing cellulite = 0.6 p

-necrotizing fasciitis = 0.4 p

-phlegmon of the dorsal side of the foot = 0.4 p

-abscess of the calf = 0.2 p

-fistulizing osteitis = 1.1 p

-deep phlegmon of the foot = 0.4 p 

**V. Infectious bone impairment ** 0.3 p

**VI. Visceralization of diabetes**

-nephropathy = 0.3 p

-retinopathy=0.3 p

-cardiac disease = 0.3 p

**VII. Emergency ** 1.1 p (operation during the first 24 h)

**VIII. Associated impairments**

-compensated = 0.2 p

-decompensated = 0.4 p

**IX. Surgical interventions for the diabetic foot in antecedents**

- No intervention = 0 p

- One intervention = 0.2 p

- Two interventions = 0.4 p

 - More than two interventions = 0.6 p

**X. Glycaemia + leukocytosis**

**Table 3:** Glycaemia + leukocytosis: assigned value

Glycaemia (mg/dl)	Leukocytes(/mm3)	Assigned Value (points)*
115-150	11,000-15,000	0.2
150-200	15,000-20,000	0.4
200-300	15,000-20,000	0.6
300-400	15,000-20,000	0.8
>400	>20,000	1
*Everything outside these intervals is evaluated at 0 p

XI. **Circulatory condition**

Ankle-brachial index 

-insufficient ABI =0-0.4

-weak ABI =0-0.4

-satisfactory ABI =0.41-0.9

-average ABI =0.41-0.9

-good ABI =0.91-1.3

The new reference values of the therapeutic-prognostic index are the following: 

- For values of less than 6 limited interventions, minor amputations are also recommended;

- For values of more than 6 major amputations, (calf and thigh) will be performed; 

## Results

Of the patients retrospectively analyzed, most are male (73.3%) and belong to the age category 60-69 years old, 2.4% are patients with diabetes of type I and 97.6% are patients with diabetes of type II.

**Table 4:** Distribution according to gender and age groups, retrospective sample

Age	Female	Male	TOTAL
20-29	1	1	2
30-39	0	1	1
40-49	6	28	34
50-59	23	99	122
60-69	34	128	162
70 and over	56	73	129
TOTAL	120	330	450

As far as the method of compensation for the sugar diabetes is concerned, 43.1% have followed a treatment with insulin and 56.9% have used oral antidiabetics, providing them with a low metabolic balancing.

31.5% of the patients retrospectively analyzed were predominantly neuropathic and 68.5% were arteriopathic and 67.3% have shown associated cardio-vascular antecedents (arterial hypertension, ischemic cardiac disease, congestive cardiac insufficiency, atrial fibrillation). 

As far as the diagnostic upon admission is concerned, the distribution, within the respective sample is as it follows: 

**Chart 1:**
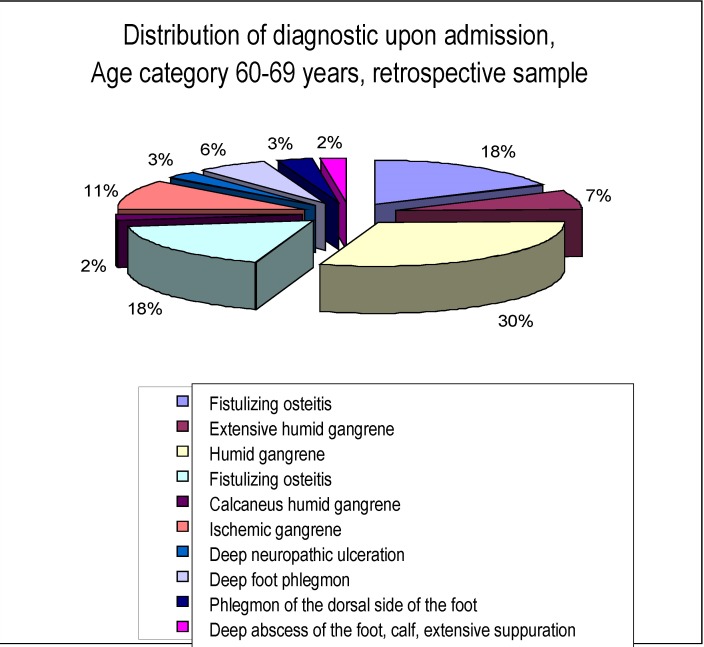
Distribution of diagnostic upon admission, Age category 60-69 years, retrospective sample

The analysis of the lot from the prospective point of view has yielded the following results: 

- Highlights an approximately equal number of patients in the age categories 60-69 years (31.7%) and over 70 years (31.9%), the majority being males (74.3%);

- 97.7% are patients suffering from sugar diabetes of the type II, the rest of 2.3% suffering from sugar diabetes type I; 

- 36.5% of the patients are neuropaths and 63.5% of the patients are predominantly arteriopaths; 

- The distribution of diagnostic upon admittance, on the age groups, with the highest number of patients is shown below: 

**Chart 2:**
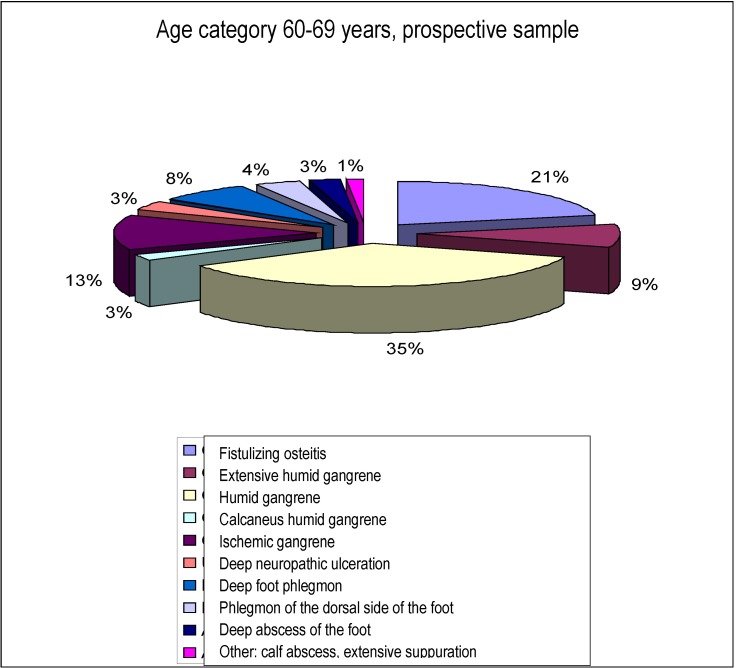
Distribution of diagnostic upon admission, Age category 60-69 years, prospective sample

**Chart 3:**
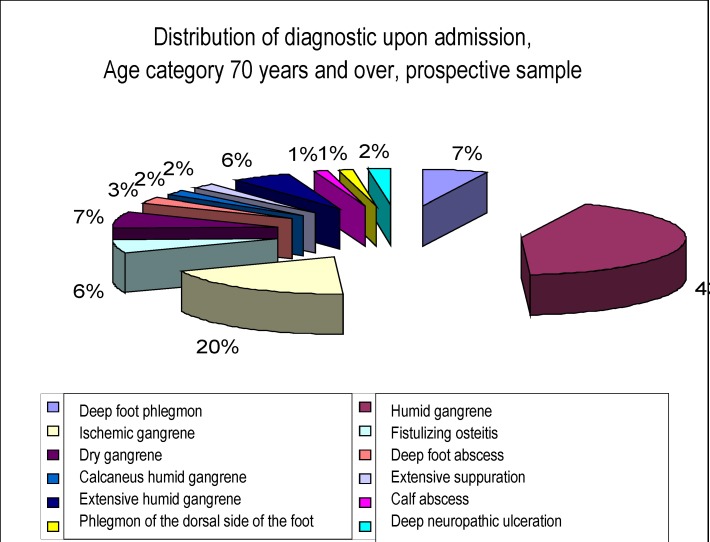
. Distribution of diagnostic upon admission, Age category 70 years and over, prospective sample

The new therapeutic-prognostic index was calculated for the retrospective lot, resulting in a concordance between the effective surgical intervention and the prognostic index of 79.4% and, in patients who were prospectively evaluated, a confirmation of the relation between the surgical intervention and the forecast by means of calculating the index at 82.6% was found.

## Discussion

Most of the patients who were analyzed from the retrospective as well as from a prospective point of view, were males, predominantly arteriopathic, treated with oral antidiabetics, with associated cardio-vascular impairments and having most often a gangrene of the toe as a diagnostic upon admission.

The usefulness of the new therapeutic-prognostic index resides in the existence of large number of invalidating surgical interventions in patients with diabetic foot lesions, due to late recognition thereof and inadequate assessment of the severity of lesions.

By extending the sample of patients who were studied, the statistical significance of the analysis increased and led to an in-depth approach of the evaluation potential of the severity of the diabetic foot lesions, determining an adequate therapeutic conduct. 

The new therapeutic-prognostic index found its usefulness especially in such medical centers in which the surgical pathology of the diabetic foot was less well known, thus avoiding repeated operations, on a ground which was already fragile due to the presence of diabetes, often found at a stage in which compensation showed a deficit.

## Conclusions

The new therapeutic-prognostic index is an effective method in establishing the surgical indication for the patient suffering from lesions of the diabetic foot, its calculation being easily done, the results being based upon the most important parameters that evaluate the conditions of the diabetic disease and its complications.
